# Can the Divide be Bridged: Overview of Life in Urban Slums in India

**DOI:** 10.4103/0970-0218.62562

**Published:** 2010-01

**Authors:** Vijay Viswanathan, Shabana Tharkar

**Affiliations:** 1 Managing Director and Head, M.V. Hospital for Diabetes and Diabetes Research Centre, (WHO Collaborating Centre for Research, Education and Training in diabetes) No-4, Main Road, Royapuram, Chennai, India. E-mail: drvijay@mvdiabetes.com; 2 Department of Epidemiology, M.V. Hospital for Diabetes and Diabetes Research Centre, (WHO Collaborating Centre for Research, Education and Training in diabetes) No-4, Main Road, Royapuram, Chennai, India

Sir,

India is getting urbanized rapidly. It is estimated that by 2030 40.6% of the country's population will be living in urban areas.(1,2) More than one-third of the urban population in India live in cities (i.e., settlements with population of more than 1,000,000).(1) The number of such cities has increased from 1 in 1901 to 35 in 2001, and continues to rise.(3) The explosive growth of Indian cities can be attributed to the opening of multinational retail firms and IT giants producing numerous opportunities and urban migration of the villagers who seek employment as daily wage skilled and unskilled labourers or domestic helps. A large proportion of these migrants end up residing in subhuman conditions in slums. The rapid growth of cities has widened the gap between the demand and supply of essential services and infrastructure, forcing people to live in crowded slums in unsanitary conditions, exposing themselves to pollution and natural calamities.

In Chennai, more than 25% of the city's total population are slum dwellers.(3) About 40% of this slum population lives along the rivers and canals of Chennai and the rest on the pavements of the city.(4) The slum population of metropolitan cities is largely neglected in terms of provision of health care facilities. We carried out this study in 2006 with the objective of exploring the living conditions and determining the health-related problems that affect the underprivileged section of the urban population of Chennai city. Data collection was done by trained health workers.

A representative sample of 900 subjects from slum tenements, selected by cluster random sampling from four zones of Chennai city, formed the study population. The mean age of the subjects was 24.3 ± 17 years. There were 326 males and 574 females. Data was collected by administering a structured questionnaire covering sociodemographic details, housing and environmental details, health problems, and behavior. The surveyed subjects were taken to a hospital for clinical examination physical and blood pressure measurements and basic blood investigations like hemogram and random blood sugar.

The results showed that poverty was very common. About 18.8% had a monthly income of Rs. 2000, 36.2% earned Rs. 2000-3000/month, and 45% had an income of Rs. 3000-8000/month. Rates of illiteracy (males: 48.2%; females: 73.9%) and unemployment (males: 46.4%; females: 65.6%) were very high. The living conditions were poor, with lack of basic amenities such as access to clean drinking water (35%) and poor sanitation facilities (41%). About 53.1% of the surveyed population lived in temporary shelters such as thatched huts or tents or on pavement/railway platforms., Among those who lived in temporary shelters, 48% did not have provision of drinking water facility and 66% did not even have toilet facilities.

The health conditions detected in the study population are as follows: Respiratory illness was present in 17.2% of the study population and 13.5% had other infections. The mean hemoglobin levels of the total population aged 20 years and above was 12.3 ± 2.4 and 11.1 ± 1.4gm/dl, for males and females respectively. Anemia was significantly higher among females of all age groups. The prevalence of anemia among females was 30%, 43% and the rates were as shown-age-group 13-19 years male: Female ratio was 17%: 30%, age-group 20-39 years, 11%: 43% and age group 40-59 years had 21% and 46% for males. The prevalence of underweight in children aged 5-10 years was extremely high as assessed by the WHO child growth weight-for-age Z scores(5) method [Table 1].

A similar study done among the children of high socioeconomic status in Chennai showed that the mean weight of children was higher than the WHO reference value, showing that large intra-urban differences exist between children from different socioeconomic backgrounds.(6)

**Table 1:** Prevalence of underweight among children aged 5-10 years as assessed by the Z score tables - weight for age, WHO growth reference

Age in years	Z-score	Male no. (%)	Female no. (%)
5	≤ −3.0 SD	3 (16.7)	8 (33.3)
(n = 42;M: 18, F: 24)	−2.9 SD to −2.0	3 (16.7)	4 (16.7)
	−1.9 SD to −1.0 SD	6 (33.3)	2 (8.3)
6	≤ −3.0 SD	6 (40.0)	3 (25.0)
(n = 27;M: 15, F: 12)	−2.9 SD to −2.0 SD	2 (13.3)	5 (41.7)
	−1.9 SD to −1.0 SD	4 (26.7)	1 (8.3)
7	≤ −3.0 SD	0.0	0.0
n = 26;M: 9, F: 17)	−2.9 SD to −2.0 SD	4 (44.4)	2 (11.8)
	−1.9 SD to −1.0 SD	2 (22.2)	2 (11.8)
8	≤ −3.0 SD	2 (20.0)	3 (27.3)
(n = 21;M: 10, F:11)	−2.9 SD to −2.0 SD	1 (10.0)	1 (9.1)
	−1.9 SD to −1.0 SD	7 (70.0)	4 (36.4)
9	≤ −3.0 SD	0.0	1 (7.1)
(n = 20;M: 11, F: 9)	−2.9 SD to −2.0 SD	2 (18.2)	4 (28.6)
	−1.9 SD to −1.0 SD	2 (18.2)	4 (28.6)
10	≤ 23.0 SD	6 (31.6)	2 (28.6)
(n = 26;M: 19, F: 7)	−2.9 SD to −2.0 SD	6 (31.6)	1 (14.3)
	−1.9 SD to −1.0 SD	6 (31.6)	3 (42.9)

The prevalence of hypertension among the adults was 21.4%, and glucose intolerance was 8.6%. Hypertension and diabetes are considered lifestyle-related disorders but, they seem to be prevalent even among the poor. It must be stressed that the major proportion of these cases remains undiagnosed. Eye disorders like defective vision, cataract, and uncorrected myopia together were present in 22.5% of the surveyed population.

One distressing factor and issue of concern was the high prevalence of child abuse, with 23.7% of the children reporting abuse in the form of violence at home or on the streets. It has long been recognized that the environment in which a person lives significantly affects his/her health. Women and children have a double impact from exposure to the environmental pollution outside as well as from the toxic fumes of stove inside their ill ventilated huts leading to detrimental effects on health.

Other Indian studies done among slum dwellers have shown similar results about poor access to drinking water and sanitation facilities(7) and that the urban poor have inadequate access to health services.(8)

To conclude, the living and environmental conditions of the study subjects were poor. Our study found a high prevalence of infections, underweight, and under-nutrition. Lifestyle-related disorders like hypertension and glucose intolerance, which were once considered ‘rich man's diseases,’ are becoming common even among the poor. Illiteracy and unemployment being the root cause of all the problems must be urgently addressed. Strategies must be formulated for improvement of nutrition and sanitation. Last but not the least, provision of good and accessible healthcare facilities can definitely bring about some improvement in their health. A holistic, multisector approach is required to develop an integrated and cost-effective method to improve the standard of living and health service delivery to the poor.

## References

[1] UN Population Division World Urbanization Prospects: The 2007 Revision Population Database.2.

[2] IBEF July 27, 007.

[3] 2001 Census, Registrar General of India.

[4] Housing and Urban Development Department Policy Note 2005-2006. Demand No.25.

[5] ttp://www.who.int/growthref/who2007_weight_for_age/en/index.html.

[6] Shabana Tharkar, Vijay Viswanathan (2009). Impact of Socioeconomic Status on Prevalence of Overweight and Obesity among Children and Adolescents in Urban India. Open Obesity Journal.

[7] Mony PK, Verghese L, Bhattacharji S, George A, Thoppuram P, Mathai M (2006). Demography, environmental status and maternal health care in slums of vellore town, Southern India. Ind J Comm Med.

[8] Sheela Prasad, Ramachandriah Health services and disease profile in Hyderabad city, a Pilot Study.

